# PReS13-SPK-1321: Expanding clinical spectrum of autoinflammatory diseases

**DOI:** 10.1186/1546-0096-11-S2-I31

**Published:** 2013-12-05

**Authors:** I Aksentijevich

**Affiliations:** 1 Inflammatory Disease Section, National Human Genome Research Institute, Bethesda, USA

## 

The term autoinflammation was initially introduced to denote a group of diseases that lacked the usual features of autoimmunity (high-titer autoantibodies and antigen-specific T cells), and were subsequently recognized as disorders of the innate immune system. Patients with autoinflammatory disorders present with chronic and recurrent bouts of systemic inflammation that are medi-ated by the cells of the innate immune system such as neutrophils and macrophages. The concept of autoinflammation started with hereditary recurrent fevers, but has grown substantially to include both monogenic and polygenic diseases. Hereditary recurrent fevers are the prototypic monogenic disorders, whereas Behçet disease, Crohn disease, gout, spondyloarthropathies, type-2 diabetes are all considered complex autoinflammatory diseases. The monogenic autoinflammatory diseases are inherited in an autosomal-recessive or autosomal-dominant fashion, and many of disease-causing mutations are found in genes that regulate the IL-1 signaling pathway. More recently, new pathways such as the IL-36, immunoproteasome, HOIL-1 deficiency, and phospholipase Cγ2 pathways have been identified in the pathogenesis of autoinflammation. Some of these new autoinflammatory diseases and relevant pathways will be discussed at the meeting. Despite major advances, a substantial number of patients have no mutations in the known autoinflammatory genes. The present challenge is how to find the as-yet undiscovered genes, considering that most cases are sporadic or occur in small families that are not suit-able for linkage analysis. New approaches and tools such as next-generation sequencing are the most likely methods to be successful. Such research might require collabora-tive studies in order to increase the number of patients presenting with a rare phenotype.

## Disclosure of interest

None declared.

